# Epidermal Growth Factor Receptor Cell Proliferation Signaling Pathways

**DOI:** 10.3390/cancers9050052

**Published:** 2017-05-17

**Authors:** Ping Wee, Zhixiang Wang

**Affiliations:** Department of Medical Genetics and Signal Transduction Research Group, Faculty of Medicine and Dentistry, University of Alberta, Edmonton, AB T6G 2H7, Canada; pwee@ualberta.ca

**Keywords:** epidermal growth factor receptor, ErBB, signal transduction, ERK, AKT, cell cycle, cell proliferation, G1

## Abstract

The epidermal growth factor receptor (EGFR) is a receptor tyrosine kinase that is commonly upregulated in cancers such as in non-small-cell lung cancer, metastatic colorectal cancer, glioblastoma, head and neck cancer, pancreatic cancer, and breast cancer. Various mechanisms mediate the upregulation of EGFR activity, including common mutations and truncations to its extracellular domain, such as in the EGFRvIII truncations, as well as to its kinase domain, such as the L858R and T790M mutations, or the exon 19 truncation. These EGFR aberrations over-activate downstream pro-oncogenic signaling pathways, including the RAS-RAF-MEK-ERK MAPK and AKT-PI3K-mTOR pathways. These pathways then activate many biological outputs that are beneficial to cancer cell proliferation, including their chronic initiation and progression through the cell cycle. Here, we review the molecular mechanisms that regulate EGFR signal transduction, including the EGFR structure and its mutations, ligand binding and EGFR dimerization, as well as the signaling pathways that lead to G1 cell cycle progression. We focus on the induction of *CYCLIN D* expression, CDK4/6 activation, and the repression of cyclin-dependent kinase inhibitor proteins (CDKi) by EGFR signaling pathways. We also discuss the successes and challenges of EGFR-targeted therapies, and the potential for their use in combination with CDK4/6 inhibitors.

## 1. Introduction

Epidermal growth factor (EGF) receptor (EGFR), also known as ErbB1/HER1, is the prototype of the EGFR family that also includes ErbB2/HER2/Neu, ErbB3/HER3, and ErbB4/HER4 [1]. Driven largely by its role in promoting cell proliferation and opposing apoptosis, the EGFR has been vilified as a proto-oncogene. New facts regarding the complex signaling network activated by the receptor tyrosine kinase are continuously emerging, highlighting the fact that there is still much to discover about the EGFR before we can optimally fine-tune effective EGFR-targeting therapies. In this review, we will examine our knowledge on how the mitogen EGF activates the EGFR and its signaling pathways. We will also explore how aberrations in the EGFR lead to oncogenesis by focusing on the role of the EGFR in cell cycle progression, including on their effect in activating Cyclin D-CDK4/6. Furthermore, we will examine the rationale behind anti-EGFR treatment options, and their possible synergisms with recently developed CDK4/6 inhibitors.

### 1.1. Brief History—Discovery

Stanley Cohen’s Nobel Prize-winning studies provided the seminal work for the EGFR field. In 1959, together with Rita Levi-Montalcini, Cohen was examining the effects of a newly discovered growth factor, the nerve growth factor (NGF), on newborn mice. Their source of NGF consisted of crude extracts prepared from mouse salivary glands. Dr. Levi-Montalcini had noticed that unexpected “side effects” were present in whole fraction preparations unrelated to the effects seen in NGF-purified fractions [2]. The responsible component was causing precocious tooth eruption and eyelid opening in the newborn mice. Cohen focused on these “side effects” and isolated the sole component [3] and termed it the epidermal growth factor [4], as it seemed to enhance the epidermal growth of animal cells in vivo [5] and in vitro [4]. Cohen soon discovered that murine EGF could enhance DNA synthesis in cultured fibroblasts from humans, which allowed him to discover the human counterpart of murine EGF [2]. The question soon turned to the mechanism of action of EGF. At the time, it was thought that peptide hormones would bind to receptors on the plasma membrane, but then be released into the extracellular environment. However, Cohen showed that EGF could bind a membrane-bound 170 kDa protein, the putative epidermal growth factor receptor (EGFR) [6,7,8]. This binding would cause the EGF and the EGFR to be internalized into the cell, which would subsequently be degraded through a lysosome-dependent manner. Using the A-431 cell line, a human epidermoid carcinoma cell type rich with EGFR, he discovered that the addition of EGF caused the phosphorylation of the EGFR. Originally identified as a threonine kinase, it wasn’t until the fact that the Rous sarcoma virus was found to encode a tyrosine kinase that Cohen’s lab correctly identified the EGFR as a receptor tyrosine kinase [9], the first of its kind to be defined.

The EGFR’s link to cancer was first recognized when the transforming v-ErbB oncogene of the avian erythroblatosis virus was found to be a mutant homolog of human EGFR [10,11]. The v-erbB oncogene was found to contain recombinations of the transmembrane and cytoplasmic domains of the EGFR [12], implicating EGFR aberrations to cancer. In addition to mutations, overexpression of EGFR was then observed to promote cancer progression, first in carcinomas [13,14], and later on in sarcomas [15], non-small cell lung cancer (NSCLC) [16] and malignant gliomas [17]. The levels of EGFR would soon be found to predict tumor grade, patient prognosis, and relapse in cancer [18,19]. In the lab, it was found that EGF addition to EGFR-overexpressing cells caused a transformation phenotype in NIH 3T3 cells that could form tumors in nude mice, largely due to its role in promoting cell cycle progression [20,21].

It is well established that the ErbB family of proteins can promote a multitude of pro-oncogenic biological processes, including cell proliferation, angiogenesis, inhibition of apoptosis, cell motility, adhesion, and metastasis. It is clear that ErbB family members represent promising targets for pharmacological inhibition. Various cancer treatment options targeting mutated or overexpressed ErbBs have reached the clinics, and tend to show efficacy and improve long term outcome. However, it has become evident that cancer cells can employ various molecular mechanisms to escape cell death mediated by anti-ErbB treatments. The characterization of these pathways has taken centre stage, in hoping that these discoveries will lead to effective combination therapies.

### 1.2. ERBB Family Members

The human ErbB gene family includes EGFR/ERBB1/HER1, NEU/ERBB2/HER2, ERBB3/HER3, and ERBB4/HER4. Being able to form homo- and heterodimers with one another, these family members can form a total of 28 different combinations with each other [22]. ERBB2 does not contain a ligand-binding domain, and no direct ligand for it has ever been identified, yet ERBB2 appears to be the preferred binding partner to its family members [23,24,25], as its dimerization arm is constitutively exposed [26]. ERBB3 does not contain a kinase domain, therefore ERBB3 homodimers possess little autophosphorylation activity, albeit some is present [27]. However, ERBB3 can still be phosphorylated and induce potent downstream signaling [25].

In *Caenorhabditis elegans*, the ErbB gene is a single receptor, with a single ligand [28]. In *Drosophila*, ErbB is made up of one receptor and four ligands [29]. EGFR pathways signaling studies in *Drosophila* have yielded tremendous information, especially regarding embryogenesis [30].

### 1.3. EGFR Expression

EGFR is mapped to chromosome 7 short arm q22, spanning 110 kb of DNA divided into 28 exons [31,32]. In normal cells, the expression of EGFR is estimated to be from 40,000–100,000 receptors per cell [33], whereas overexpression of more than 10^6^ receptors per cell is observed in cancer cells [34].

EGF regulates its own receptor, as it increases EGFR RNA expression [35] by stimulating the expression of ETF (EGFR-specific transcription factor) [35,36]. Other proteins that modulate the *EGFR* promoter include E1A [37], Sp1 [36,38], and AP2 [39]. The interaction between DNA topoisomerase I and c-JUN has also been shown to regulate *EGFR* gene expression [40].

### 1.4. Physiological Role of EGFR

Almost all cell types possess ErbB family members, with the exception of hematopoietic cells [41]. EGFR family genes are critical to the normal embryogenesis of vertebrates [42]. Null mutations of any of the ErbB genes in mice cause embryonic or perinatal lethality [43,44]. The specific phenotype depends on the genetic background of the mouse, but also on the ErbB family member that is knocked out. The lethality of EGFR null mice have been shown to be due to abnormalities in organs including in the brain, skin, lung, and gastrointestinal tract, as well as to the renewal of stem cells [45,46]. ERBB2 null mice die from cardiac trabeculae dysfunctions, but also exhibit malformations in motor nerves and sensory ganglia [47,48,49]. ERBB3 null mice suffer from defective cardiac formation [50] and severe neuropathies [51]. ERRB4 knockout mice die from defective trabeculation in the heart, similar to ERBB2 knockout mice, and also exhibit improper hindbrain-derived cranial neural crest cell migration [52,53,54]. EGFR also plays roles in rat embryonic skin maturation, hair follicle development, hair cycling [55], and corneal development [56].

In adolescence, EGFR family genes play key roles in mammary ductal development. Female mice with a T743G substitution that impairs tyrosine kinase activity fail to develop proper mammary glands, due to defective ductal growth, causing their pups to die from malnutrition [57,58,59].

EGFR activity remains high in most parts of mature CNS [60]. However, in mouse and rat astrocytes, EGFR is present in high levels in developing astrocytes, but becomes absent in mature astrocytes [61,62]. Interestingly, CNS disorders including ischemia, tumor development, and neurodegenerative diseases can re-upregulate EGFR in astrocytes, in a response termed reactive astrogliosis [63,64].

### 1.5. EGFR Structure

The EGFR is synthesized as a 1210 residue precursor that is cleaved at the N-terminal to result in the mature 1186 residue transmembrane EGFR [11]. From N-terminal to C-terminal, the EGFR consists of (1) an extracellular ligand binding and dimerization arm (exons 1–16), (2) a hydrophobic transmembrane domain (exon 17), and (3) the intracellular tyrosine kinase and C-terminal tail domains (exons 18–28) [43]. Here, we will describe the structure and function of each domain in the EGFR.

The extracellular region of the EGFR is composed of 621 amino acids and is subdivided into four domains, I (amino acids 1–133, exons 1–4), II (amino acids 134–312, exons 5–7), III (amino acids 313–445, exons 8–12), IV (amino acids 446–621, exons 13–16). Domains I and III are leucine-rich fragments that participate in ligand binding. Domain II forms homo- or hetero-dimers with the analogous domain of family members. Domain IV can form disulfide bonds to domain II, and links to the TM domain. Domains II and IV do not make contacts with the ligand, and are cysteine-rich regions.

The TM domain is a 23 amino acid long hydrophobic single pass membrane structure, that anchors the receptor to the membrane [65]. It is 23 amino acids long, from Ile622 to Met644 [66]. The EGFR TM domain has been suggested to play a role in dimerization, as the N-terminal region of the TM helices have been hypothesized to contact during dimerization [67]. The role of the TM domain has been better studied in ERBB2, as mutations in the ERBB2 TM domain augment dimerization [68]. Furthermore, the ERBB2 TM may also be involved in aligning the intracellular domains through rotational twisting [69].

The intracellular domain is 542 amino acids long, and includes the flexible juxtamembrane segment (~40 aa), the tyrosine kinase domain (amino acids 690–953, exons 18–24), and the C-terminal tail (amino acids 954–1136, exons 25–28) [70]. The tyrosine kinase domain can be divided into an N-lobe (mainly a β-sheet structure) and a C-lobe (a mainly α-helical structure), with an ATP-binding site located between the two lobes [71]. Transautophosphorylation relies on the interaction of the N-lobe of one receptor to the C-lobe of the other [72]. The kinase domain also contains lysine residues that are the primary sites of receptor ubiquitination. The C-terminal tail includes various tyrosine residues, which when phosphorylated, allow the anchoring of a variety of intracellular proteins to the activated receptor. These proteins then participate in the signal transduction relay.

### 1.6. EGFR Mutations

EGFR mutations occur at mutational “hotspots” in the extracellular region, the kinase domain, and the C-terminal tail [1]. Certain types of cancers appear to favor certain locations for their mutations. For example, most glioblastomas appear to harbour aberrations to the ectodomain, whereas non-small cell lung cancers (NSCLCs) almost exclusively harbour kinase domain mutations [1,73]. Colorectal cancers less commonly harbour EGFR gene mutations [74]. Rather, around 50% of colorectal cancers have been reported to contain EGFR gene amplification, with a three- to five-fold increase in copy number [75]. The EGFR is also overexpressed in 40–80% of NSCLC [76]. Most EGFR mutations and truncations promote the constitutive activation of EGFR by stabilizing ligand-independent dimerization with ERBB family receptors [77,78]. Furthermore, some mutations allow the receptor to escape downregulation by endocytosis [79]. Mutations in the EGFR transmembrane region are rarely seen.

EGFR ectodomain oncogenic alterations often cause the loss of the inhibitory regulatory domains for dimerization. The most famous EGFR ectodomain mutant is the viral EGFR homologue, v-ERBB, which completely lacks the ectodomain, and exists primarily in dimers [10]. In addition, the EGFR variant EGFRvIII arises from the genomic deletion of exons 2–7, and occurs in approximately 20% of glioblastomas [73,79]. Interestingly, EGFRvIII displays ligand-independent signaling, but has low constitutive activity. The low constitutive activity is enough to impart cancer cells with increased signaling, however its growth advantage is due to the fact that these receptors are not downregulated by endocytosis [79,80].

In the kinase domain, the most commonly seen EGFR point mutation is L858R, and makes up approximately 45% of mutations in the tyrosine kinase domain [81,82]. Termed a “classical” activating mutation, the L858R mutation of the activation loop confers 50-fold more kinase activity and higher K_M_ for ATP than wild type EGFR [83,84]. Molecularly, crystallographic analysis suggests that the leucine to arginine substitution causes the activation loop to “flip out,” which destabilizes the auto-inhibited conformation normally found in non-ligand bound EGFR, essentially leading to a stabilized active conformation [72,85]. Another classical class of activating mutations in the kinase domain are the various *EGFR* exon 19 in-frame deletions, commonly observed in NSCLC [86]. Another kinase domain mutant, T790M, is often referred as the “gatekeeper residue,” and is notorious for conferring resistance to pharmacological EGFR tyrosine kinase inhibitors in addition to increasing EGFR phosphorylation levels [87,88]. While the mechanism of resistance of T790M is not yet known, the bulkier methionine side chain may provide steric hindrance to TKI binding [89]. T790M is located in exon 20, and other insertions in exon 20 have also been found to confer resistance to TKIs [86].

## 2. Signal Transduction

The first studies on the EGF in animals showed that it stimulated epidermal proliferation and keratinization [5]. We now know that the EGF can stimulate cell proliferation, cell differentiation, cell growth, migration, and inhibit apoptosis. It has been shown that the addition of EGF to HeLa cells activates the EGFR to cause the global phosphorylation of 2244 proteins at 6600 sites [90]. Furthermore, it was shown that EGF stimulation causes significant differences in expression of 3172 genes and 596 proteins in human mammary epithelial cells (HMEC) [91]. Another study showed that the ERBB signaling network is estimated to encompass 122 proteins and 211 interaction [92]. Even without factoring in other types of post-translational modifications, it is clear that the effects of the EGF on the cell are profound and wide-ranging. The signal transduction mediated by the EGFR is extremely complex. It begins with the EGFR being activated by one of its ligands, leading to receptor dimerization, the transphosphorylation of the C-terminal tail, and finally the propagation of the signal through various intricate signaling pathways to induce the expression of new genes. EGFR mutations and truncations can impart the EGFR with ligand-independent signaling, which lead to the upregulation of various pro-oncogenic processes, including chronic cell cycle proliferation.

### 2.1. Ligand—EGF

Human EGF is a 6 kDa protein made up of 53 amino acids. Physiologically in humans, various organs regulate their innate EGF concentrations [93]. For example, EGF is found at high concentrations (50–500 ng/mL) in bile, urine, milk, and prostate fluid, at medium concentrations (5–50 ng/mL) in tears, follicular fluid, sperm, and seminal plasma, and at low concentrations (1–2 ng/mL) in plasma, serum, and saliva (mice differ in that their saliva are high in EGF) [93,94]. The sources of EGF from the human body have been documented previously [95]. EGF is implicated in the morphogenesis of teeth, brain, reproductive tracts, skin, gastrointestinal tracts, in cardiovascular differentiation and function, epithelial regeneration, and corneal epithelia (reviewed in [96]). However, no disorders arising from EGF deficiency have been identified, likely due to the presence of other EGFR ligands [97].

Aside from the EGF, six other EGFR ligands have been described. These include transforming growth factor-α (TGF-α), amphiregulin (AREG), epiregulin (EREG), betacellulin (BTC), heparin-binding EGF-like growth factor (HB-EGF), and epigen (EPI). EGF, TGF-α, and amphiregulin are specific ligands only for the EGFR [98]. TGF-α is composed of 50 amino acids, and displays 35–40% homology with EGF [41]. BTC, HB-EGF, and EREG show dual specificity for both EGFR and ERBB4 [99]. Notably, the neuregulins (NRGs) can bind both ERBB3 and ERBB4, or only ERBB4, depending on the subclass [100,101,102,103].

Each ligand appears to activate the EGFR in the same way: through ligand binding, receptor dimerization, receptor transautophosphorylation, and the recruitment of signaling proteins or adaptors. In addition, all EGFR ligands induce EGFR internalization and trafficking to early endosomes [104]. Despite these similarities, the activation of the EGFR by different ligands has been shown to cause distinct downstream biological activities [24,97]. The exact mechanism by which each ligand brings about distinct biological effects is unclear, however some theories have been put forward. One theory is that each ligand induces preferred dimer pairs within the EGFR family, which leads to a distinct signal. It was shown that although EGF and NRG can both stimulate ERK1/2, EGF acts primarily through EGFR and ERBB2 heterodimers to preferentially stimulate PKC, whereas NRG acts primarily through ERBB2 and ERBB3 heterodimers to preferentially stimulate AKT [105]. Another theory examines the contribution of the type of ligand to the fate of the receptor. Following endocytosis, endocytic sorting generally targets receptors bound with EGF for lysosomal degradation or recycling, with HB-EGF and BTC for lysosomal degradation, with TGF-α and EPI for receptor recycling, and with AREG for fast and slow EGFR recycling [104,106]. The differential sorting may be due to the different pH sensitivities of each ligand-receptor combinations. A study reported that although EGF and TGF-α bind the EGFR with similar affinities at relatively neutral pH (7.4), a drop in pH to 6 (to mimic acidification in EEs) led to the dissociation of TGF-α from the EGFR that allowed the receptor to be recycled back to PM, whereas the EGF-EGFR complex remained stable and led to its trafficking to degradation components [107]. The study also showed that recombinant EGF mutants with different affinities for the EGFR altered the endosomal sorting of the EGFR. Another study showed that the recycling of TGF-α-bound EGFR back to the PM effectively prevents the downregulation of the EGFR, thus allowing more pronounced and sustained EGFR signaling [108]. Therefore, since the ligand affects the location of the EGFR within the cell, each ligand is likely able to create distinct signaling patterns that tunes the biological response respectively. Lastly, it is hypothesized that each ligand may cause different conformations of dimerized receptors, resulting in differential accessibilities of substrates to the C-terminal tail [109]. The exact mechanism of the differential signaling is still a subject of research.

Most growth factors act in a paracrine fashion, where the growth factor produced from a cell targets a neighboring cell. However, examples of autocrine mechanisms have been documented. Originally elaborated by Sporn and Roberts [110], this dangerous situation puts the cell in charge of its own cell division. Many viruses take advantage of this autocrine signaling to turn on the cell’s DNA replication program in order to duplicate its own genome. For example, the EGFR is the target of sarcoma virus-transformed mouse fibroblasts, which encodes EGF-like molecules to stimulate its own growth [111].

### 2.2. EGFR Dimerization

For full EGFR activation, ligand binding and EGFR dimerization are crucial. However, it was a matter of controversy which process occurred first. It is now well accepted that for the activation of the EGFR, EGF-binding to each EGFR monomer precedes EGFR dimerization. Originally observed by Yarden and Schlessinger [112,113], the ability to visualize crystal structures have shown that mechanistically, prior to ligand binding, domain II is folded into domain IV via disulfide bonds, in a “tethered” conformation that auto-inhibits dimerization [26,114]. EGF binding to the EGFR monomers at domains I and III promotes a domain rearrangement to expose the dimerization arm in domain II, leading to a stabilized “open” or “extended” conformation. [26,114]. Dimerization of domain II is followed by rearrangements in the transmembrane domain that also leads to rearrangements in the juxtamembrane segment. The intracellular juxtamembrane segment then forms interactions with the kinase domain that are important for the formation and stability of the EGFR dimer [115]. The EGFR dimer is an asymmetric EGFR dimer pair, in that the C-terminus of the activating kinase inserts into the active site of the receiving kinase, so that this allosteric interaction can activate the receiving kinase, resulting in transautophosphorylation [72]. This is different to other RTKs, where dimerization induces a conformational change that induces each receptor monomer to activate itself.

There is evidence that preformed unliganded EGFR dimers do exist [116,117,118], however their precise structure and function are still controversial. Using quantum dot-based optical tracking of single molecules, researchers found that these preformed temporally-transient dimers were observed to be stabilized together at the dimerization arms, and were enriched at the cell periphery in regions of high actin and EGFR expression [119]. Some researchers have concluded that these preformed EGFR dimers may be primed to bind EGF [116,117]. However, others have argued against this, showing that preformed extracellular dimers of the EGFR are structurally heterogeneous [120,121]. Moreover, isothermal titration calorimetry measurements showed that pre-dimerized EGFRs did not have enhanced ligand binding, whereas ligand binding could promote dimerization [120]. However, these studies were conducted using only the extracellular region of the EGFR that was engineered to self-dimerize with the addition of a dimerizing F_c_ domain or of a dimeric leucine zipper. The characterization of the interactions between EGFR domains remains difficult since many experiments are carried out predominantly with isolated domains, either extracellular, TM, or intracellular. As such, it is difficult to understand the synergy between each domain throughout ligand activation [122].

The site of EGFR dimerization is considered “receptor-mediated”, in that the ligand does not make a direct contribution to the dimerization interface [22]. Each EGFR monomer binds an EGF ligand, and the receptors interact through domain II to form the dimer interface. This is in stark contrast to other RTKs that use the ligand as a bridge between receptors to form the dimer. The other extreme is “ligand-mediated” dimerization, where the ligand is itself a dimer and bridges together two receptors by their ligand-binding fragments. For example, two nerve growth factor molecules bridge together two TrkA receptors without the receptors ever making contact. Two intermediatory forms of dimerization have also been described. The first is the formation of a ligand-mediated dimer that includes receptor contacts, such as for c-KIT and stem cell factor. The second, such as in the FGFR, includes a combination of bivalent ligand binding, direct receptor-receptor contacts, but also requires an accessory molecule [22].

Although EGFR has been proclaimed as a prototypical, its unique “receptor-mediated” mechanism of dimerization and its ability to transautophosphorylate make it anything than such. These subtle differences lead to approaches by which pharmaceutical targeting of the EGFR could take advantage of these facts.

### 2.3. Signaling Pathways

**Figure 1:**
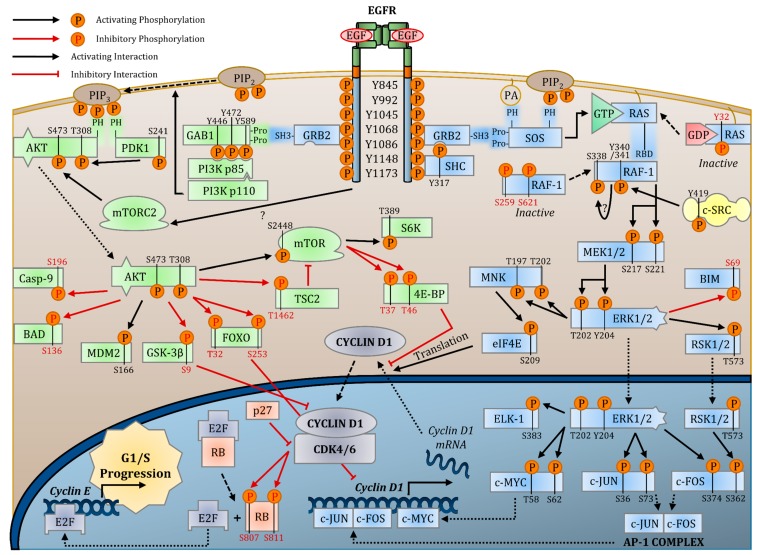
Epidermal growth factor receptor (EGFR) signaling pathways leading to G1/S cell cycle progression activated by EGF activation. Depicted are the RAS-RAF-MEK-ERK MAPK and PI3K-AKT-mTOR pathways. EGF activation of the EGFR induces receptor dimerization and transphosphorylation of the C-terminal domain. The phosphorylated C-terminal domain binds SHC and GRB2, along with PLC-γ1 at Y992 (not pictured). The GRB2 SH3 domain recruits the proline-rich domains of SOS or GAB1 to initiate ERK MAPK or AKT signaling respectively. SOS is also recruited to the plasma membrane (PM) by the interaction of its PH (pleckstrin homology) domains with PIP_2_ (phosphatidylinositol-4,5-bisphosphate) and PA (phosphatidic acid). SOS catalyzes the conversion of GDP to GTP of RAS. Active RAS uses its RAS RAF-binding domain (RBD) to recruit RAF-1. RAF-1 is activated by dephosphorylation and phosphorylation events, and activates MEK1/2. Activated MEK1/2 activates ERK1/2. ERK1/2 has various cytoplasmic and nuclear targets, which aid in the transcription and translation of Cyclin D1. For example, ELK-1 transcribes the *c-FOS* gene (not pictured), and the protein product together with c-JUN make up the AP-1 complex. The AP-1 complex as well as c-MYC induce the transcription of *CYCLIN D1*. On the other hand, the receptor-bound GRB2 also binds GAB1. GAB1 recruits the p85 regulatory subunit of PI3K, which binds the p110 catalytic subunit. PI3K converts PIP_2_ into PIP_3_ (phosphatidylinositol-3,4,5-triphosphate). PIP_3_ recruits AKT, and is phosphorylated and activated by PDK1 and mTORC2. AKT has many phosphorylation substrates, including various inhibitory phosphorylations to proteins that negatively regulate CYCLIN D1 activity such as GSK-3β (normally induces CYCLIN D1 degradation) and FOXO (normally represses *CYCLIN D1* transcription). Furthermore, AKT inhibition of TSC2 allows the activation of mTOR, which inhibits the inhibitor of translation 4E-BP, thus allowing eIF4E-mediated translation of *CYCLIN D1*. Increased levels of CYCLIN D1 correlates with increased CDK4/6 activity, which phosphorylates RB. Phosphorylated RB releases the E2F transcription factor, which participates in the transcription of Cyclin E and leads to G1/S progression. The CDK inhibitor protein p27 inhibits CYCLIN D-CDK4/6 activity. However, activated ERK, c-MYC, AKT, all inhibit p27 activity. Importantly, the activated ERK MAPK and AKT pathways also inhibit various pro-apoptotic proteins, including BIM, Caspase-9, and BAD.

The signal transduction pathways activated by the EGFR comprise the most important reasons the EGFR has been studied to such lengths. The powerful capacity of each of the multitude of pathways under the EGFR’s control to drive cell proliferation and resist apoptosis has formed a strong motivation for their cancer-related researches. Large strides have been accomplished in elucidating the pathways involved in mediating EGFR activity. Current research largely focuses on the crosstalk and compensation between pathways, so to elucidate how cancer cells develop resistance to pharmacological inhibition of pathways and how to properly intervene. The metabolic alterations mediated by these pathways are also of focus. Here, we will review the activation mechanisms by which the EGFR activates the ERK MAPK, AKT-PI3K, and PLC-γ1-PKC pathways, focusing on the molecular interactions between each protein in the pathways (Figure 1). We will focus on the important domains and amino acids that help propagate the signaling pathways.

#### 2.3.1. EGFR Transautophosphorylation

Of the four family members, the EGFR signals to the largest number of unique signaling pathways, including the ERK MAPK, PI3K-AKT, SRC, PLC-γ1-PKC, JNK, and JAK-STAT pathways. As these pathways are inter-linked, the activation of the EGFR actually stimulates an entire signaling network associated with a wide number of outcomes, such as cell proliferation, growth, differentiation, migration, and inhibition of apoptosis. Many proteins within the EGFR’s signal transduction network have been the subject of pharmaceutical targeting in malignancies, illustrating the potency of the receptor.

Binding of EGF to the EGFR leads to the transphosphorylation of various tyrosine residues on the intracellular C-terminal tail. The tyrosine residues phosphorylated by EGF addition to cells include Y703, Y920, Y992, Y1045, Y1068, Y1086, Y1148, and Y1173. In addition to these autophosphorylated sites, there are also residues that are phosphorylated by other kinases, which interestingly appear downstream in the EGFR-activation cascade. For example, Y845 is phosphorylated by c-SRC [123], and T654 is phosphorylated by PKC [124]. Regardless, the newly phosphorylated tyrosine residues serve as docking sites for proteins harboring phosphor-tyrosine-binding residues, such as those with Src Homology 2 (SH2) and phosphotyrosine binding (PTB) domains [125]. Other important domains in the EGFR’s signal transduction include SH3 (binds proline-rich), 14-3-3 (binds phosphoserine), bromo (binds acetylated lysine), and PH domains (binds phosphorylated inositides).

#### 2.3.2. RAS-RAF-MEK-ERK MAPK Pathway

The RAS-RAF-MEK-ERK MAPK pathway may be the most important pathway in mediating the biological response of the EGFR. Bonafide proto-oncogenes RAS and RAF reside in this pathway. A major target of cancer therapy is MEK. ERK MAPK (mitogen-activated protein kinase) interacts with over a hundred substrates to initiate a wide array of physiological and pathological responses, including growth, proliferation, differentiation, migration, and inhibition of apoptosis [126,127]. Substantial bodies of evidence have supported the pharmacological targeting of this pathway for cancer treatment. Here, we will first briefly outline the domains and amino acid residues that mediate the binding of a protein to the next protein in the signaling cascade. Then we will explore each protein in more details.

Overview of RAS-ERK MAPK Pathway

Following receptor transphosphorylation, the activated EGFR’s Y1068 and Y1086 residues bind directly to GRB2 (growth factor receptor binding protein 2), by its SH2 domain from residues 60–158 [128,129,130]. Additionally, the activated EGFR Y1148 and Y1173 residues can also recruit SHC (Src homology and collagen) [131,132,133] preferentially through it PTB domains, but also through its SH2 domain [134]. Notably, mutations to SHC of Arg175 in the PTB domain and of Arg397 in the SH2 to lysine prevent binding activity [134]. Regardless, these two adaptors link the ligand-activated EGFR to complex intracellular biochemical pathways. Upon binding to the EGFR, SHC is phosphorylated at Y317, which becomes a binding site for GRB2 [132,135]. SHC is also phosphorylated at Y239/240 by SRC, with this phosphorylation being important for SHC association with GRB2 [136]. GRB2’s two flanking SH3 domains bind to the proline rich carboxy-terminal tail in SOS1 (son of sevenless 1), which includes residues 1069 to 1138 [137,138,139,140,141]. SOS is a guanine nucleotide exchange factor (GEF) for RAS small guanosine triphosphatase (GTPase), and activates RAS by inducing it to exchange GDP to GTP [142]. RAS can then interact with the RAF-1 Ras-GTP-binding domain (RBD), which contains amino acids 55–131 [143]. RAF-1 is a complex protein, but phosphorylation of its Ser338 and Tyr341 residues have been shown to be important binding sites for MEK1/2 [144,145,146,147]. RAF-1 directly activates MEK1/2 by phosphorylation at serine residues 217 and 221 [148]. MEK (mitogen-activated protein kinase kinase-MAPKK) 1/2 are a rare class of tyrosine and threonine/serine dual-specificity kinases that activate ERK1/2. MEK1/2 phosphorylates the Thr-Glu-Tyr motif in the ERK1/2 activation loop, at T202 and Y204 [149]. ERK1/2 then phosphorylates multiple substrates to induce various biological response.

SHC and GRB2

Receptor transphosphorylation forms binding sites for both SHC and GRB2 adaptor proteins. Complexes that contain SHC have been reported to be better at activating RAS than those without SHC (i.e., direct binding of GRB2 to EGFR) [150]. Recruitment of GRB2 to the plasma membrane (PM) by SHC or EGFR occurs within two minutes of EGF treatment [151]. GRB2 was originally discovered for its role in EGF-induced SOS recruitment to the PM [128]. However, GRB2 can also recruit various other proteins to the PM. For example, GRB2 can bind phospholipase D, which hydrolyzes phosphatidylcholine to form the important secondary messenger phosphatidic acid (PA) and choline [152]. Importantly PA catalyzed by PLD2 forms an important binding site for subsequent SOS membrane recruitment [153]. The authors also found that although the SOS PH domain is recruited to the PM by PIP_2_, it is more efficiently recruited by PA. GRB2 can also bind CBL and CIN85, two important proteins for EGFR trafficking [154,155,156]. CBL is a ubiquitin ligase that ubiquitinates the EGFR and mediates its internalization and lysosomal sorting [93]. Although CBL can bind directly to the EGFR at Y1045, GRB2-mediated CBL recruitment to the EGFR is extremely important to properly respond to varying doses of EGF to mediate the proper endocytic response [157].

SOS and RAS

As mentioned, SOS recruitment to the PM requires GRB2, PIP_2_, and PA. In addition, non-substrate RAS can form a binding site for SOS recruitment to the PM, which can potentiate the important GTP exchange activity of SOS for RAS by 500-fold [158]. Moreover, SOS can interact with the PLC-γ1 SH3 domain to help mediate RAS activation [159]. The exchange of GDP with GTP by SOS causes a conformational change in RAS, which turns on RAS activity. RAS is turned off by its intrinsic GTPase activity, which catalyzes its GTP to GDP, a process that can be accelerated by GTPase activating proteins (GAPs), such as NF1 (Neurofibromin 1) [160]. It was recently reported that RAS activity is inhibited by SRC-mediated phosphorylation at Y32, which reduces binding of RAS to RAF-1 and increases the GTPase activity of RAS, effectively shutting off two facets of RAS activity [161]. On the other hand, the phosphatase SHP2 has been identified by the same group to dephosphorylate pY32, thus implicating SHP2 as a direct activator of RAS activity [162]. Therefore, the latest model for the RAS activation cycle is as follows [163]. RAS activation requires SOS-induces GTP loading to RAS, along with SHP2 dephosphorylation of Y32 activate RAS. Activated RAS then engages with its downstream effectors. To turn off RAS, SRC phosphorylates Y32, causing the displacement of RAF, further followed by GTP hydrolysis to inactivate RAS.

Active RAS has the ability to activate three major downstream effectors. These include RAF-1 (also known as c-RAF), PI3K, and RalGDS (Ras-like guanine nucleotide-dissociation stimulator) [164,165,166]. The ability of RAS to activate these major pathways indicates why RAS-activating mutations or mutations within the RAS pathways are among the most common causative genetic alterations in cancer. It is estimated that up to 30% of all human tumours carry some mutation in the *RAS* genes [167]. However, despite over 30 years of research, RAS has been described as undruggable. Typically, to inhibit the activity of kinases, the ATP-binding pocket usually represents a target of pharmacological blocking with relatively high specificity. However, in the case of RAS, a functional GTP-catalyzing pocket is required to shut off its activity, precluding the site from pharmacological inhibition. Therefore, specifically targeting RAS is a difficult endeavor. Furthermore, the activation of 5% of total RAS molecules in the cell is estimated to be sufficient to induce full activation of ERK1/2, thus requiring an extremely efficient inhibitor to sufficiently inhibit RAS activity [168]. While the direct pharmacological inhibition of RAS is still being explored, alternative approaches to targeting RAS regulators such as PHLPP, SHP-2, and NF-2 have also been proposed [162,169,170].

RAF

RAS itself does not fully activate RAF. Rather, RAS initiates RAF activation by translocating it to the plasma membrane, where additional membrane-localized activation events occur. RAS recruits RAF through the RBD region. In addition to this site, the RAS cysteine-rich domain (CRD, amino acids 139–184) has been shown to be important for the efficient activation of RAF-1 [171,172,173,174]. RAF is a serine-threonine protein kinase with complex regulation [175]. It has been estimated that activating mutations of RAF occur in ~7% of all human cancers, with gain-of-function B-RAF mutations being largely associated with melanomas [176,177]. RAF is composed of an N-terminus regulatory domain and a C-terminus kinase domain. Only a small pool of RAF-1 is activated in ligand-stimulated cells [168]. Activated RAF-1 is phosphorylated at S338 and Y341, which are about 20 amino acids upstream of the ATP-binding domain in the regulatory region [178]. It has been reported that oncogenic RAS predominantly leads to S338 phosphorylation, and that activated SRC predominantly phosphorylates Y341 [179]. RAF S338 phosphorylation is induced by the addition of growth factor, however the molecular mechanism is unclear. It has been proposed that RAF S338 phosphorylation occurs by auto-phosphorylation [145]. RAF S338 may also be phosphorylated by PAK3 (p21-activated kinase 3) [180], although this has been debated [181]. PAK3 is activated by plasma membrane-localized small GTP-binding proteins CDC42 and RAC, which are also downstream targets of RAS. In addition, PAK1 has been shown to phosphorylate S338, but likely in a growth factor-independent context [182,183,184]. In addition to S338 and Y341, the phosphorylation of S471 in the catalytic loop, and of T491 and S494, have been reported to stimulate RAF-1 activity [185,186]. The phosphorylation of two other sites of RAF-1, S259 and S621 is inhibitory. Both pS259 and pS621 are bound by 14-3-3, which keeps RAF-1 in an inactivate conformation [187,188]. The phosphorylation of S259 is catalyzed by AKT [189].

EGF treatment recruits 14-3-3-associated RAF-1 to the PM and leads to dissociation of 14-3-3 from RAF-1 [190]. It has been reported that RAS binding to RAF-1 displaces 14-3-3 from the N-terminus, but not the C-terminus of RAF-1 [190,191]. pS259 is dephosphorylated by PP2A (protein phosphatase 2A), and is essential for RAF-1 membrane association [192,193]. The significance of the pS621 site is controversial, although it appears RAS-binding to the site is necessary for RAF-1 kinase activity [194]. The requirement of the site has been difficult to study however, as mutations to S621 cause the mutant RAF-1 to lose kinase activity [195]. Although 14-3-3 appears to inhibit RAF-1 activity, its reversible association with RAF-1 appear necessary for its activation via RAS-dependent interactions [194,196]. Furthermore, serine residues 29, 43, 289, 296, 301, and 642 have been reported as ERK-catalyzed phosphorylation sites, which are associated with feedback inhibition [197]. RAF-1 has also been shown to dimerize with itself or family member B-RAF to activate its kinase activity [198]. Interestingly, so far only cancers with mutant B-RAF V600G can be effectively treated with therapeutics [199]. Interestingly, in HeLa cells, the concentrations of RAS, RAF, and downstream proteins MEK and ERK have been estimated at 400, 13, 1400, and 960 nM respectively, and revealed that RAF inhibition could be mostly effective in inhibiting this signaling pathway [200].

MEK and ERK

MEK1 and MEK2 are activated by RAF-1, its only known substrates [201]. Furthermore, the only known MEK1/2 substrates are ERK1/2, with ERK1/2 only known to be activated by MEK1/2 [127,199]. Constitutively activate MEK1 greatly reduces growth factor dependency for cell proliferation, yet MEK1/2 is not commonly found to be mutated in solid tumors, [202,203].

ERK1 and ERK2 are serine/threonine kinases that always appear to be activated together [204]. Unlike the narrow substrate specificity of RAF and MEK, ERK has over one hundred downstream cytoplasmic and nuclear substrates [205]. In the cytosol, it can activate RSK1 (p90 ribosomal S6 kinase 1) by phosphorylation at T573, a site located in the C-terminal kinase domain activation loop of RSK1 [206]. Phosphorylation of RSK1 at T573, as well as S221, S363, and S380 have been identified to be important for its activity [207]. The kinase PDK1 (phosphoinositide-dependent protein kinase 1), of the PI3K-AKT pathway, can also activate RSK [208]. RSK translocates to the nucleus to activate c-FOS and SRF [209]. Activated ERK itself also translocates to the nucleus to activate ternary complex factor (TCF) transcription factors, which play a major role in the induction of immediate early genes (IEGs) [210]. IEG products include c-FOS and c-MYC, which induce late-response genes, to promote various phenotypes associated with ERK signaling [211]. Moreover, nuclear ERK can activate ELK-1, ETS, SP-1, and c-JUN [136,212,213]. ERK can phosphorylate ELK-1 at S383, S389, and S422, residues located at the C-terminal transcriptional activation domain [214,215]. c-FOS and c-JUN make up components of the AP-1 (activator protein 1) complex, which functions as a transcription factor that binds to the *CYCLIN D1* promoter to activate transcription of the important G1 driver [216,217] (see Section 3). c-FOS is phosphorylated at S374 by ERK1/2 and at S362 by RSK [218]. c-JUN is phosphorylated at S63 and S73 by ERK1/2 in mouse fibroblasts [219]. EGF-activated ERK1/2 has also been found to both stabilize and induce the expression of FOXC1, a transcription factor with implications in triple-negative breast cancer [220,221]. ERK also negatively regulates apoptosis by phosphorylation of the pro-apoptotic BH3-only protein BIM (BCL2-like 11) on S69, which induces BIM ubiquitination and proteasome degradation. ERK is also known to regulate pyrimidine synthesis, chromatin remodeling, ribosome synthesis, and protein translation (reviewed in [222]).

#### 2.3.3. PI3K-AKT-mTOR Pathway

The PI3K-AKT-mTOR signaling cascade controls metabolism, proliferation, cell size, survival and motility. In cancer, this pathway is often hyper-activated due to activating mutations to EGFR family members, PI3K, AKT, and downregulation of the famous tumour suppressor PTEN, which antagonizes PI3K activity. The PI3K-AKT-mTOR pathway is also dysregulated in diabetes, autism, and aging [223].

PI3K was discovered in the 1980s by the Cantley group [224]. It was shortly found to be activated by EGF stimulation [225]. There are three classes of PI3K, Class I, II, and III, which defer in structure, regulation, and function [226]. Here, we will only discuss Class I PI3K, the major downstream effector of EGFR. Class I PI3K are further subdivided by subclass: subclass IA (PI3Kα, β, and δ) is activated by receptor tyrosine kinases, and subclass IB (PI3Kγ) is activated by G protein coupled receptors [227]. PI3K is comprised of a regulatory p85 subunit that mediates binding to the receptor, and a catalytic p110 domain that phosphorylates the 3-OH group of the membrane lipid phosphatidylinositol-4,5-bisphosphate (PIP_2_) to generate phosphatidylinositol-3,4,5-triphosphate (PIP_3_) [224,228,229]. The newly formed PIP_3_ is a potent secondary messenger and is the predominant mediator of PI3K activity [230]. The strong signaling potential of PIP_3_ is highlighted by the fact that the PI3K antagonist PTEN, which dephosphorylates and limits the activity PIP_3_. is frequently inactivated in cancer [231]. PIP_3_ links the lipid kinase activity of PI3K to the network of downstream signaling pathways, including the PH (pleckstrin homology) domain-containing serine/threonine kinase AKT/PKB. PI3K also indirectly stimulates the production of phosphatidylinositol-3,4-bisphosphate (PtdIns(3,4)P_2_), which can also recruit PH domain-containing proteins, including AKT. Other than AKT, cells contain 50–100 downstream effectors of PI3K [232]. Other notable effector proteins of PI3K that contain PH domains are the RHO, RAC, RAS, ARF, and GAB1/2 proteins [232].

The recruitment of PI3K differs between ErbB family members following ligand stimulation. PI3K binds directly with ERBB3 and ERBB4 [233,234], but indirectly with ERBB1 and ERRB2 [235]. The p85 SH2 domain recognizes the phosphorylated Tyr-X-X-Met motif only present in ErbB3 and ErbB4 [233]. However, indirect interactions for ERBB1 and ERBB2 to PI3K are mediated by the adaptor protein GAB1 (GRB2-associated binder), which contains the canonical PI3K-binding sites [236]. However, the majority of GAB1 likely binds to the EGFR through GRB2, by the interaction between the GAB1 proline-rich domain with the GRB2 SH3 domain [237,238,239]. This causes the tyrosine phosphorylation of GAB1, at Y446, Y472, and Y589, which are binding sites for the recruitment of the p85 subunit of PI3K [236]. RAS can also bind and recruit PI3K by the p110 subunit, and a point mutation at K227E of PI3K has been shown to block p110 subunit binding of activated RAS [166,240,241]. In addition, CBL’s Tyr371 and Tyr611 residues also bind to the p85 regulatory subunit of PI3K, allowing CBL to act as an adaptor to bring PI3K to the EGFR [235]. Moreover, PI3K dephosphorylation of Y688 on p85 by SHP1 (SH2 domain-containing phosphatase) is activating, whereas phosphorylation of the residue by c-SRC is inhibitory [242].

AKT (also known as Protein Kinase B, or PKB) is a serine/threonine kinase with a wide variety of substrates that impact cell survival, proliferation, metabolism, protein synthesis, growth, and migration. It is activated by a dual regulatory mechanism, requiring its translocation to the PM and phosphorylation at two conserved residues. Activated EGFR stimulates AKT translocation to the PM by activating the PI3K-induced formation of PIP_3_. AKT binds to PIP_3_ through its PH domain [243]. In addition, it has been found that EGF-induced ubiquitination of AKT through the Skp2-SCF E3 ligase also recruits AKT to the PM [244]. Once localized at the PM, AKT is phosphorylated at T308 and S473. T308 phosphorylation is necessary and sufficient for AKT activation, however maximal activation is achieved by phosphorylation at S473 [245,246]. PDK1 phosphorylates AKT at T308, a residue located in the kinase domain [247]. Active PDK1 is autophosphorylated at S241 [248]. Phosphorylation at the tail domain residue S473 is mediated by mTORC2, through a little understood process that involves PI3K activity [223,249]. AKT activation is suppressed by the phosphatase PHLPP (PH domain leucine-rich repeat protein phosphatase), which dephosphorylates S473 [250,251], and T308 by PP2A [252].

There are three members of the AKT family: AKT1, AKT2, and AKT3. AKT2 and AKT3 have 81% and 83% amino acid homology to AKT1 respectively [232]. AKT isoform protein and mRNA levels have been characterized in various cell lines [253]. AKT1 and AKT2 are broadly expressed, whereas AKT3 is only expressed in the brain, heart, and kidney [254,255], which may explain why AKT3 is less well studied than the first two isoforms. There is no consensus on the role of each isoform in cells, due to the controversial pool of data regarding their function. AKT1 has been found localized to the cytoplasm and nucleus, AKT2 to mitochondria, and AKT3 to the nucleus and nuclear membrane [253], although their localization may differ based on the cell type [256]. AKT2 appears to be the main AKT responsible for glucose metabolism and the induction of apoptosis, which supports its mitochondrial role. In fact, Akt2 null mice seem unable to maintain glucose metabolism whereas Akt1 null mice maintain normal metabolism, but are smaller compared to normal mice [257,258,259]. Many Akt1 null mice suffer embryonic lethality, suggesting Akt2 and Akt3 cannot fully compensate for Akt1 activity [254]. In fact, it has indeed been observed that an Akt isoform will not compensate for the loss of another isoform, as Santi and colleagues found that siRNA ablation of one or two Akt isoforms did not significantly alter the subcellular localization of the remaining Akt isoform [253]. In terms of cancer, some studies have found that AKT2 may be the primary AKT for mediating PI3K-mediated metastatic processes [260,261], whereas others have concluded AKT1 to be as important [262,263,264]. Interestingly, some studies suggest opposing roles for the two isoforms. For examples, Irie and colleagues found that AKT2 promoted cell invasion, whereas AKT1 inhibited cell migration [261]. Similarly, Arboleda and colleagues showed that AKT2 overexpression promoted adhesion and invasion in human breast cancer cell lines, whereas AKT1 overexpression did not [260]. Another study found that AKT1 was important for lung tumor growth, whereas AKT2 inhibited this growth in mice [265]. Regardless, it appears both AKT1 and AKT2 aberrations can play tumourigenic roles. Overexpression of AKT1 has been reported in gastric and breast cancers [266,267]. Furthermore, the gain-of-function E17K somatic mutation of AKT1 occurs in breast, colorectal, and ovarian cancers [268]. AKT1 also appears to mediate tumour development in *Pten* haplodeficient mice [269]. On the other hand, AKT2 overexpression has been reported in ovarian, pancreatic, and colorectal cancers, and in hepatocellular carcinomas [270,271,272,273]. Genetic amplification of *AKT2* is prominently observed [270,274,275].

Regarding the regulation of AKT isoforms under the EGFR, all isoforms appear to be under the control of PI3K upon EGF stimulation in various esophageal cancer cells [276]. However, depending on the cell line, the AKT isoforms are differentially activated in a little understood RAS-dependant manner. EGF stimulation does not appear to change AKT expression levels [253]. In the absence of serum or growth factors, EGFR overexpression does not appear to constitutively activate AKT, however ERBB2 overexpression has been shown to do so in breast cancer cells [276,277].

AKT mediates its wide range of physiological responses through the activation or deactivation of several downstream proteins. Here, we discuss its role in mediating cell survival, in activating mTOR, and in metabolism. The role of AKT in cell proliferation, including its inhibition of GSK-3β (glycogen synthase kinase-3 beta), is discussed in Section 2.5.1.

In terms of cell survival, AKT functions in an anti-apoptotic manner by directly phosphorylating components of the cell death machinery. AKT phosphorylates the pro-apoptotic BAD (Bcl-2-associated death promoter) protein at S136, which inactivates BAD and prevents it from binding and inhibiting the anti-apoptotic BCL-X_L_ protein [278]. AKT also inhibits the catalytic activity of caspase-9 by phosphorylation at S196 [279], as well as the activity of FOXO1 by phosphorylation at T32 and S253 [280]. FOXO1 downstream gene targets include pro-apoptotic proteins BIM and the FAS ligand [281]. AKT also phosphorylates MDM2 at S166 and facilitates its translocation to the nucleus, where it ubiquitinates and downregulates p53, the well-known tumour suppressor [282,283,284].

One of the most important AKT pathways is to signal to mTOR (mammalian target of rapamycin). mTOR refers to two distinct complexes: mTORC1 is made up of raptor, GbL/mLST8, and negative regulatory subunits PRAS40 and DEPTOR, whereas mTORC2 contains rictor, mSin1, and Protor, GbL/mLST8 and DEPTOR [285]. mTORC1 is well known to regulate cell growth and autophagy. The mTOR receives stimulatory signals from growth factors through RAS and PI3K, as well as from nutrient inputs through amino acids, glucose, and oxygen availability [286]. Growth factor stimulation activates AKT, ERK, and RSK, which all phosphorylate and inactivate TSC2, an inhibitor of mTORC1. TSC1 and TSC2 suppress the activity of RHEB (Ras-like GTPase), which are needed to activate mTORC1 [287]. AKT directly phosphorylates TSC2 on five residues, including Ser939, Ser981, Ser1130, Ser1132 and Thr1462 [288,289]. ERK1/2 directly phosphorylates TSC2 on S664 [290]. Therefore, AKT, ERK, and RSK signaling converge to deactivate TSC2 and activates mTORC1, which phosphorylates 4E-BP (eurakyotic initiation factor 4E-binding protein) and S6K (p70 S6 kinase) [291]. These two effectors of mTORC1 have tremendous impacts on protein synthesis and cell growth, as 4E-BP is an inhibitor of translational inhibition and S6K is an activator of translation [223]. In terms of important phosphorylation sites, mTOR can be phosphorylated at S2448 by AKT and correlates with mTOR activity, although S2448A mutation still allows mTOR to activate S6K [292]. Furthermore, mTOR S1261 and S1415 phosphorylation have been reported to increase mTORC1 activity [293,294]. Downstream signaling occurs by phosphorylation of 4E-BP at T37 and T46, and of S6K at T389. mTOR activation also leads to the increased synthesis of CYCLIN D1, HIF1 (hypoxia-inducible factor 1), and growth factors including VEGF [295,296,297].

In the past decade, more research has been done to understand the role of the PI3K-AKT-mTOR pathway in the control of cell metabolism and glycolysis in cancer. In normal cells, high oxygen levels favor the use of oxidative phosphorylation over glycolysis, as it is a more efficient mechanism for generating ATP. Tumour cells however continuously use glycolysis even at high oxygen levels, a term coined aerobic glycolysis and also the principle behind the phenomenon known as the Warburg effect [298]. Although aerobic glycolysis is less efficient than oxidative phosphorylation at generating ATP, cancer cells continue using it, as it also synthesizes macromolecules that support cancer growth, such as ribose-5-phosphate, acetyl-CoA, and NADPH [298]. AKT is one of the main drivers of the Warburg effect, as it increases glucose uptake through the upregulation of glucose transporters [299,300]. In addition, various components of the PI3K-AKT-mTOR pathway also coordinate the uptake of nutrients, including glucose, glutamine, nucleotides, and lipids, to better support the enhanced proliferation and growth needs of cancer cells [301]. Activating mutations to the PI3K-AKT-mTOR pathway essentially reprogram the cell’s metabolism, therefore targeting the unique metabolic dependencies of cancer cells through the PI3K-AKT-mTOR network may provide much therapeutic gain.

#### 2.3.4. PLC-γ1-PKC Pathway

Prior to the discovery of PI3K, interest on PIP_2_ focused on the results of its receptor-mediated hydrolysis by phospholipase C (PLC) [232]. Thirteen PLC isozymes have been described so far, and they are grouped within six classes based on their function and regulatory activation mechanisms [302]. Notably, the *Plcg1* null knockout mouse is embryonic lethal at E9.0, in part due to the impairment of vasculogenesis [303,304]. PLC-γ1, the protein encoded by *Plcg1*, has been shown to upregulate cell migration and invasion in vitro and in vivo, including upregulating metastasis in cancer [305,306].

PLC-γ1 binds directly to activated EGFR at Y992 and Y1173 using its SH2 domain [303,307,308,309]. PLC-γ1 can also be recruited to the PM using its PH domain by binding PIP_3_ formed by PI3K in response to EGF stimulation [310,311]. Phosphorylation of PLC-γ1 at Y472, Y771, Y778, Y783, and Y1254 have been shown to be important for its activity [312,313]. Once recruited to the vicinity of the PM and activated, PLC-γ1 hydrolyzes PIP_2_ into free intracellular 1,4,5-triphosphate (IP_3_) and diacylglycerol (DAG), two important secondary messengers. IP_3_ binds to IP_3_-receptors at the endoplasmic reticulum to induce intracellular calcium release. Calcium release converges with the DAG pathway, as both DAG and Ca^2+^ activate protein kinase C (PKC). Phosphorylation at the activation loop of the kinase domain of PKC by PDK1 is necessary for its activation, and the specific residues that are phosphorylated have been characterized [314]. For example, PKCα, one of the PKC isoforms activated by DAG, is phosphorylated at T497 to mediate its activation [315,316]. PKC has a host of cellular substrates, including EGFR, RAF-1, H-RAS, p21, GSK-3β, RHOA, BAD, and BCL-2 [314]. Interestingly, PKC-dependent phosphorylation of EGFR T654 blocks EGF-induced EGFR activation [317]. Another substrate is phospholipase D (PLD). PKC phosphorylates PLD at S2, T147, S561, which mediates a large signaling network within the EGFR network [318,319,320]. Elevated PLD activity has been shown to contribute to fibroblast transformation by synergizing with EGFR and SRC [321,322] Phospholipase D hydrolyzes phosphatidylcholine to form phosphatidic acid (PA) and choline [152]. PA can interact with proteins such as RAF, RAC, PIP5K, mTOR, and S6K (reviewed in [323]).

#### 2.3.5. SRC

The first virus ever identified to cause cancer encoded a viral isoform of c-SRC (also known as pp60src). The isoform, discovered in 1911 by Francis Peyton Rous, was named v-SRC, as it induced sarcomas in chickens [324]. v-SRC lacks an important Y527 (Y530 in human c-SRC) inhibitory site. In inactive c-SRC, Y527 is phosphorylated and induces an inhibitory intramolecular loop between itself and the SRC SH2 domain [325,326,327]. CSK (c-Src kinase) has been implicated as the kinase for Y527 [328,329]. SHP-1, SHP-2, PTPγ, and PTP-1B have been implicated as phosphatases for Y527 [330,331,332,333]. Furthermore, auto-phosphorylation at Y416 (Y419 in human c-SRC) displaces the pY416-containing activation loop from the catalytic cleft, thereby allowing SRC to gain access to substrates [334]. c-SRC was the first protein discovered to have tyrosine kinase activity [335,336,337]. Today, 11 nonreceptor tyrosine kinases make up the Src Family Kinases (SFKs), including c-Src, Fyn, Yes, Blk, Yrk, Frk, Fgr, Hck, Lck, and Lyn (reviewed in [338]). c-SRC, YES, and FYN are ubiquitously expressed in most tissues [339].

c-SRC is widely implicated in various aspects of EGFR signaling. For example, it can directly phosphorylate EGFR Y845, RAF Y341, SHC1, clathrin, and CBL [340]. It is not clear how EGFR-mediates c-SRC activation, however EGF stimulation indeed leads to c-SRC activation [341]. EGFR and ERBB4 both possess binding sites for SRC [109]. This activation is reportedly mediated by RAS and RAL [342]. EGFR and SRC have been reported to functionally synergize to form more aggressive cancers [341,343,344]. c-SRC may help activate STAT (signal transducer and activator of transcription) transcription factors in a JAK-independent manner [345]. c-SRC phosphorylates EGFR at Y845, which is not an autophosphorylation site [346]. However, Y845F mutation does not prevent c-SRC interaction to EGFR, nor does it affect ERK MAPK signaling [334,341]. Rather, Y845 has been suggested to regulate the autonomous lateral propagation of EGFR signals, which is the propagation of EGFR signals from one EGFR molecule to another without requiring binding of the ligand, potentiating EGFR kinase activity [347]. Phosphorylation of Y845 has been suggested as a diagnostic marker for various cancer treatments, including for NSCLCs [348,349]. Interestingly, pY845 is targeted by two phosphatases, PTP1B and TCPTP [350,351].

Since SRC and EGFR appear to cooperate to increase tumorigenicity, dual inhibition of SRC and EGFR has been proposed, such as in head and neck squamous cell carcinoma and colorectal cancer [352,353]. However, as mentioned in Section 2.3.2, SRC negatively regulates RAS by phosphorylation at Y32, therefore SRC inhibition leads to increased RAS activity. In fact, EGF stimulation of SRC/YES/FYN triple knockout MEFs does not induce the phosphorylation of RAS as Y32, and as such exhibit increased RAS-RAF-1 interactions [161].

#### 2.3.6. Nuclear EGFR Signaling

Several lines of evidence have shown that stimulation with EGF, as well as H_2_O_2_, UV, therapeutic agents, or ionizing radiation cause the EGFR to translocate to the nucleus, with nuclear EGFR signaling playing roles in cell proliferation, tumor progression, DNA repair [354,355,356,357,358,359,360]. High levels of nuclear EGFR have been found in various types of cancers [361,362,363], and nuclear EGFR signaling has been reported to mediate radio-resistance and chemo-resistance, such as to ionizing radiation and cisplatin [364,365,366].

Nuclear EGFR appears to be the full-length receptor [354]. The mechanism by which the EGFR translocates to the nucleus has been studied, but is still far from clear. It is suggested that the initial internalization of EGFR from the plasma membrane share similar features to clathrin-mediated endocytosis, in that the translocation is dependent on both clathrin and dynamin in cells treated with 100 ng/mL EGF [367]. Furthermore, similar to the majority of clathrin-mediated and non-clathrin-mediated forms of EGFR endocytosis, following internalization, the EGFR that is destined to the nucleus appears to first sort into endosomes, as nuclear EGFR has been shown to co-localize with endosomal markers [368]. However, there is no established mechanism for the translocation of endosomal EGFR to nucleus. Recent evidence suggests that EGFR is transported from endosomes to the Golgi, where coat protein complex I (COPI)-mediated retrograde transport later sends the EGFR to the endoplasmic reticulum [369]. For entry into the nucleus, much attention has been paid to the nuclear localization signal (NLS) present within the EGFR, that allows the EGFR to complex with importin-β and to bind with the nucleoporins of nuclear pore complexes [368].

In the nucleus, the EGFR supports gene regulation by acting as a transcriptional co-activator. it has been shown that nuclear EGFR aids in the transcription of important cell cycle progression mediators, including *CYCLIN D1* and c-*MYC*, among other proto-oncogenes [354,370]. The interaction of nuclear EGFR to the *CYCLIN D1* gene promoter has been better studied, and has been shown to require EGFR interaction with two proteins, Mucin-1 (MUC-1) and RNA helicase A (RHA) [371,372]. In this way, nuclear EGFR signaling represents another way by which the EGFR promotes cell cycle progression, highlighting the breadth and redundancy of the EGFR signal transduction network in cancer progression.

### 2.4. EGFR-Targeted Therapies

The EGFR and its signaling pathways play key roles in oncogenesis. Two major classes of EGFR-targeted therapies have been developed. The first class of therapy developed includes humanized monoclonal antibodies against the EGFR extracellular domain, designed to block ligand binding or to mediate its downregulation [373]. The second class includes tyrosine kinase inhibitors (TKIs). TKIs are ATP mimetics that bind to the receptor’s kinase pocket, which excludes ATP and prevents signal transduction [374]. Current US FDA-approved EGFR monoclonal antibodies include Cetuximab (Erbitux) and Panitumumab (Vectibix), and approved TKIs include Erlotinib (Tarceva), Gefitinib (Iressa), and Lapatinib (Tykerb), with the latter being a dual EGFR/HER2 inhibitor. These drugs have been approved for the treatment of non-small-cell lung cancer (gefitinib and erlotinib), metastatic colorectal cancer (cetuximab and panitumumab), head and neck (cetuximab), pancreatic cancer (erlotinib), and breast (lapatinib) cancer [375]. For example, gefitinib is efficacious as first-line treatment of metastatic NSCLC with EGFR exon 19 deletions or the L858R mutation [376]. Typically, EGFR inhibitors are used as monotherapy or in combination with cytotoxic chemotherapy or high-dose radiation.

Anti-EGFR therapy generally improves survival in EGFR-positive cancer patients [377]. TKIs are effective against cancer cells with EGFR L858R mutation and exon 19 deletion [376]. These two mutations account for >90% of known activating EGFR mutations [81,378]. However, certain challenges still remain for the effective use of EGFR inhibitors. EGFR-positive tumours may be resistant to EGFR inhibitors, therefore screening for biomarkers that negatively predict response to anti-EGFR therapy has been suggested. The response to current anti-EGFR therapy can be modified by factors such as: the type of somatic EGFR mutations, EGFR gene amplification, increased autocrine EGFR ligands, and mutations in EGFR signaling pathway proteins ERBB2, K-RAS, B-RAF, PI3KCA, PTEN, and BIM (reviewed in [375,379]). However, whether tumours with these different factors benefit from anti-EGFR therapy is still confusing and uncertain [380]. For example, increased EGFR expression, typically determined by immunohistochemistry, was shown to not correlate with response to EGFR inhibitors [381,382], whereas another studied showed a positive correlation [383]. It has been speculated that these controversial results are due to the variation in EGFR antibodies used in immunohistochemistry, and that a standardized testing method be established [380]. For the common glioblastoma mutation EGFRvIII, the picture is a bit more clear, as currently approved EGFR-targeted treatments do not appear to be efficacious (reviewed in [79]). Alternative targeted therapies against EGFRvIII have been developed, including the vaccine Rindopepimut and the monoclonal antibody mAb806 (also known as ABT-806). Rindopepimut did not pass Phase III clinical trials and was discontinued in 2016. ABT-806 has passed Phase I [384], and an antibody-drug conjugate based on mAb806 called ABT-414 has also been advanced to Phase II (trial identifier NCT02573324). In terms of mutations to EGFR-pathway proteins, the efficacy of anti-EGFR therapy has been best studied in cancers with K-RAS mutations, with the consensus that these cancers will display primary resistance to EGFR inhibitors [385,386,387,388,389,390]. The FDA now requires an accompanying PCR diagnostic test for K-RAS prior to the prescription of cetuximab or panitumumab for colon cancer.

In addition to the challenge of implementing bio-markers for predicting response or resistance to anti-EGFR therapy, another challenge faced by the use of EGFR inhibitors is that the duration of the response is often limited and many tumours invariably develop resistance [379]. Secondary EGFR mutations may occur following TKI treatment that circumvent the initial benefits of the inhibitor. For example T790M mutations are the most common form of acquired resistance to TKIs [87,88]. Third generation EGFR TKIs are being developed to target T790M, including Brigatinib, HM61713, osimertinib, and rociletinib [391]. In addition to T790M mutations, secondary activating mutations within EGFR signaling pathways, such as mutations in the aforementioned *K-RAS* or in *PI3K* can activate a signaling arm of EGFR regardless of EGFR activation status, rendering the EGFR inhibitor less effective. Also, epithelial to mesenchymal transition (EMT) has also been shown to frequently accompany the acquisition of resistance to TKIs, although the mechanism of the transition is still under investigation [392,393,394,395,396]. Lastly, skin toxicity remains a common side effect that frequently causes the discontinuation of the drug [397,398]. Other common side effects to EGFR inhibitors also include perturbations to the colon and cornea [399,400,401].

Inhibition of EGFR mostly causes cell cycle arrest in G1 prior to DNA synthesis [402,403,404]. The G1 arrest appears to be due to the upregulation of p27^KIP1^ activity [402,403,405,406,407,408,409,410,411]. As this is partly cytostatic, combinations with DNA-damaging agents enhances anti-EGFR therapy. Pharmacological inhibition of EGFR is highly beneficial to radiotherapy, as ionizing radiation can actually stimulate EGFR and activate its cryoprotective signaling cascades. Other mechanisms of action by which EGFR inhibition decrease tumour growth include the inhibition of angiogenic activity by reducing the secretion of vascular endothelial growth factor (VEGF), decreasing cell invasion and metastasis by inhibiting the production of matrix metalloproteinase, and through the promotion of apoptosis in EGFR-addicted cancer cells (reviewed in [412]).

TKIs inhibit EGFR phosphorylation, which effectively downregulate ERK and AKT phosphorylations [413]. Many cancer cells that first respond to TKIs and EGFR antibodies later acquire resistance by upregulating MEK and ERK signaling [414]. For example, colorectal cancers often acquire resistance to cetuximab and panitumumab treatment [382,415]. The combination of EGFR inhibitors with inhibitors of the ERK1/2 or PI3K-AKT-mTOR pathway has been explored to increase the efficacy of EGFR therapies. MEK1/2 is a convergence point of aberrant activation from potent upstream signaling proteins, and due to its narrow substrate specificity, makes it a favorable target for therapeutical intervention. Two direct inhibitors of MEK, trametinib and cobimetinib, have been approved by the FDA for melanoma treatment [416,417]. The combinatorial treatment of MEK and EGFR inhibitors appears to overcome the acquired resistance to the EGFR inhibitors [415,418]. MEK inhibition in EGFR or ERBB2-driven cancers however is complicated, as it can lead to higher PI3K/AKT activation. This is because inactivated MEK no longer activates ERK, which normally participates in the negative regulation of RTKs by phosphorylating EGFR and ERBB2 at the inhibitory T669 and T677 sites respectively [419]. Furthermore, the combination of a MEK inhibitor with EGFR and PI3K-AKT-mTOR pathway inhibitors is of great interest, although cell toxicity is an issue [420,421,422].

Therapeutic inhibition of the PI3K-AKT-mTOR pathway has been of intense study, and many potential inhibitors are currently under investigation for cancer. No PI3K or AKT inhibitors have reached the bedside so far, although three mTOR inhibitors have been approved by the FDA [423]. It has been shown that the therapeutic inhibition of the PI3K/AKT/mTOR pathways leads cancer cells to upregulate RTK activity. A mechanism by which AKT inhibition activates RTKs has been well described for the insulin receptor (IR). Here, mTOR-activated S6K inhibits IRS-1 (insulin receptor substrate 1), the adaptor protein that connects IR to PI3K, thereby negatively regulating PI3K signaling [424]. However, inhibition of EGFR family members by PI3K signaling does not rely on IRS-1. Instead, upregulation of EGFR, ERBB2, and ERBB3 in AKT-inhibited cells occurs through the de-repression of FOXO-dependant transcription of the receptors [425,426,427,428,429]. Furthermore, inhibition of the PI3K pathway also upregulates ERBB3 through an eIF4E-dependant mechanism [430]. These mechanisms of RTK upregulation when challenged with PI3K pathway inhibition maintains receptor activity, which produce more PIP_3_ to compensate for a loss of downstream signaling. Therefore, simultaneous treatment with an RTK inhibitor an PI3K-AKT-mTOR inhibitor has been used, with more favorable responses in vitro and in mice [431,432]. mTOR inhibition is also attractive since it may avoid the side effects associated with inhibition of PI3K-AKT, which have broader biological functions [433]. Importantly, targeting the PI3K-AKT-mTOR network can result in tumour resistance due to MYC activation [434]. Furthermore, another mechanism underlying tumour resistance to mTOR inhibition is the upregulation of autophagy [435].

### 2.5. Effects of EGFR Signaling on Cell Cycle Progression

The biological response of EGFR signaling is mediated by the activation of transcription factors. An important role of these transcription factors is to induce the production of CYCLIN D1, which forms a complex with CDK4/6 to initiate cell cycle progression. This EGFR-driven process of chronic proliferation represents one of the important facets behind EGFR-mediated oncogenesis. In this section, we will examine the mechanisms by which the EGFR initiates cell cycle progression, including its upregulation of CYCLIN D, and downregulation of cyclin-dependent kinase inhibitor proteins (CDKis). We will also examine the rationale for combining CDK4/6 and EGFR inhibitors, as this combination has been of recent interest [436].

#### 2.5.1. EGFR Activation of the CDK4/6-CYCLIN D Complex

The best described role for the EGFR is to drive cells through G1, largely by inducing the activation of the CDK4/6-CYCLIN D complex. A pulse of EGF stimulation during early G1 and another at late G1 are sufficient to drive cells past the restriction point (R point), the instant at which the cell commits to the completion of the cell cycle [437,438,439]. In cancers, activating mutations to the EGFR are correlated with higher expression levels of CYCLIN D [440,441,442,443,444,445]. These types of cancers also have lower patient prognostic values. EGFR-induced activation of the ERK and PI3K-AKT pathways plays a crucial role in the induction of CYCLIN D, which cascades into E2F-mediated progression past G1 and the R point. More specifically, CDK4/6- CYCLIN D phosphorylates RB at Ser807 and Ser811, causing the release of E2F, and free E2F helps transcribe key genes for the transition of G1/S, including *CYCLIN E* and *CYCLIN A* [446,447]. CYCLIN E binds CDK2 and further phosphorylates RB to complete its inactivation which drives cells to pass the R point and trigger S-phase entry [448]. Constitutively active oncogenic EGFR uses these pathways to continuously drive cells past the R point to promote hyperplasia.

The ERK pathway induces CYCLIN D1 expression through its activation of the AP-1 complex. Transcriptionally, ERK induces the expression of immediate early genes (IEGs), including c-FOS and c-JUN [211]. Post-translationally, ERK activates RSK, which activates c-FOS [209]. Nuclear ERK also directly activates c-JUN [213]. The activated c-FOS and c-JUN form the AP-1 complex, which binds to and primes the *CYCLIN D1* promoter for transcription. Sustained ERK activation is required for progression of G1 into S, as transient ERK activation due to insufficient doses of growth factor does not lead to S-phase progression [449]. This has been proposed to be due to a poorly understood AP-1-dependant manner [449].

Concomitant PI3K-AKT signaling also upregulates Cyclin D1 activity. AKT signaling induces the transcription of *c-FOS* [450,451]. Active FOXO1 (also known as FKHR) transcription factors repress *CYCLIN D1* expression [452]. However, AKT phosphorylates FOXO1 at T32 and S253, inhibiting FOXO1 and allowing the induction of *CYCLIN D1* expression [281,453]. AKT also upregulates CYCLIN D1 activity through the inactivation of GSK-3β (glycogen synthase kinase-3 beta). GSK-3β is normally active in quiescent cells, and downregulates CYCLIN D1 activity in two ways. First, active GSK-3β causes CYCLIN D proteolysis by phosphorylating it at T286, which mediates its nuclear export and degradation [454]. In the same way, GSK-3β can mediate the degradation of another cyclin, CYCLIN E, by phosphorylation at S380 [455]. Secondly, active GSK-3β represses the transcription of IEGs, including AP-1, preventing the activation of the *CYCLIN D1* promoter [456]. In response to EGF, GSK-3β is phosphorylated by AKT at S9 and inactivated, allowing the transcription of *CYCLIN D1* and the stabilization of both CYCLIN D1 and CYCLIN E [457,458]. Lastly, mTOR-mediated CYCLIN D1 upregulation has been shown to be due to increased *CYCLIN D1* mRNA translation as a result of 4E-BP1 inhibition, with the activation of eIF4E also being important [297]. The activation of eIF4E results from ERK phosphorylation of MNK1/2 (MAP kinase-interacting kinase 1/2) at T197 and T202, which then phosphorylates eIF4E at S209 [459,460].

Since CYCLIN D activates CDK4/6 to drive G1 progression, selective CDK4/6 inhibitors have been of intense recent research, driven by the availability of highly specific CDK4/6 inhibitors that also exhibit low patient toxicity and high tolerability [436,461,462,463]. These include the oral drugs palbociclib, abemaciclib, and LEE011, with both palbociclib and LEE011 having been recently approved for breast cancer in combination with letrozole, an aromatase inhibitor. CDK4 gene amplification was reported to be detected in 16% of breast cancers [464]. In addition to these cancers, CDK4/6 inhibition should be particularly actionable for tumours whose pathways hyper-activate CDK4/6. As such, the combination of CDK4/6 inhibitors with EGFR, ERBB2, MEK, or PI3K inhibitors have been of great interest [436,440,465]. Recently, the combination of CDK4/6 and EGFR inhibitors has been shown to be effective in blocking tumor resistance in mice [436].

#### 2.5.2. EGFR Inhibition of CDKi Activity

The inhibition of EGFR in various cancers with EGFR inhibitors, including monoclonal antibodies or tyrosine kinase inhibitors, lead to G1/S arrest. Multiple lines of research suggest that the cause of EGFR inhibitor-induced G1/S arrest is the upregulation of p27^KIP1^ activity [402,403,405,406,407,408,409,410,411]. p27^KIP1^ inhibits CYCLIN D1-CDK4/6 activity, thereby preventing cells from progressing to S phase [466]. Active p27^KIP1^ also inhibits CYCLIN E-CDK2, further inhibiting exit from G1 [467]. p27^KIP1^ has also been reported to induce apoptosis, although the mechanism has not been described [468,469]. Interestingly, p27^KIP1^ also downregulates *EGFR* transcription [470,471]. As EGFR inhibitors strongly upregulate CDKi protein activity, the combination with CDK4/6 inhibitors likely provides a potent mechanism of G1/S arrest. There is much evidence that EGFR-mediated pathways ERK MAPK and AKT both suppress CDKi activity.

Many studies have shown that ERK1/2 contributes to p27^KIP1^ down-regulation [472,473,474,475]. For example, it was shown that ERK1/2 phosphorylates p27^KIP1^ and promotes its degradation [475]. The inhibition of MEK by PD098059 increased the stability of p27^KIP1^ [474]. Interestingly, ERK1/2 activation must be sustained until late G1 for progression into S-phase [449], which may be important for sustaining p27^KIP1^ down-regulation. c-MYC, a commonly-mutated downstream effector of ERK, both activates *CYCLIN D1* transcription and inhibits p21^CIP1^ and p27^KIP1^ activity, demonstrating the central role of c-MYC in G1/S progression [476,477].

Active AKT inhibits CDKis p21^CIP1/WAF1^ and p27^KIP1^, by phosphorylation at Thr145 and, Thr157 and Thr198 respectively [478,479]. AKT also inhibits FOXO1, which normally induces the transcription of p27^KIP1^ and p21^WAF1^ [480,481]. Furthermore, GSK-3β inactivation by AKT downregulates p27^KIP1^ levels, as GSK-3β normally represses p27^KIP1^ activity by both inhibiting *p27^KIP1^* gene transcription and its protein interaction with CDK2 [482].

As the ERK and AKT pathways are inhibited by EGFR inhibitors, the subsequent upregulation of CDKis may provide synergy with CDK4/6 inhibitors to provide stronger G1/S arrest. In addition, the inhibition of these EGFR pathways allows the activation of pro-apoptotic proteins, including BIM, CASPASE-9, and BAD [222,278,279]. Therefore, this combination may effectively induce G1/S-arrested cancer cell death. However, this combination would need to effectively eliminate cancer cells before they demonstrate classical signs of resistance to EGFR inhibitors, including the *de novo* upregulation of EGFR signaling pathways (Section 2.4), as these would downregulate CDKis, as well as oppose CDK4/6 inhibition through the increased levels in CYCLIN D1. Therefore, a thorough ability to inhibit these pathways may hold the key to enhancing the efficacy of CDK4/6 inhibitors, and the continued effort to effectively control resistance to EGFR inhibitors, whether through the use of newer generation EGFR inhibitors, or with RAS-ERK or PI3K-AKT-mTOR pathway inhibitors may prove fruitful.

## 3. Conclusions

The EGFR is at the head of one of the most important signaling pathways to mammalian cell physiology, and to oncogenesis. As this review has shown, tremendous amounts of research has been conducted on each of the many components of the EGFR. The sum of our knowledge from this vast array of research is allowing us to create even more effective drugs or drug combinations to target cancers with EGFR aberrations. In addition to understanding these signaling pathways in cancer, the spatial and temporal regulation of the EGFR remain understudied. For example, spatially, EGFR internalization by endocytosis results in EGFR signal attenuation, and has been studied by many groups [93,483,484,485,486]. Understanding how to target the EGFR to endocytosis and lysosomal degradation may present an avenue to downregulate EGFR levels. Furthermore, EGFR endocytosis may be used to internalize cytotoxic agents specifically to EGFR overexpressing cells, such as through the use of EGFR monoclonal antibodies conjugated to various drugs [487,488]. In addition to endocytosis, although the EGFR has been well-studied temporally for its role in G1, its function throughout the rest of the cell cycle—S, G2, and mitosis—remains rarely studied. For example, the EGFR has been seldom studied during mitosis, yet a differential regulation of its signaling and endocytosis is apparent [489,490,491,492]. Further investigations of EGFR function throughout the rest of the cell cycle are warranted.
